# Effect of Dapagliflozin on Indices of Atrial Myopathy in Rheumatic Heart Disease

**DOI:** 10.1016/j.jacasi.2026.04.042

**Published:** 2026-07-08

**Authors:** Jamal Yusuf, Abhishek Nagar, Ankur Gautam, Vimal Mehta, Mohit D. Gupta, Ankit Bansal

**Affiliations:** Department of Cardiology, G.B. Pant Institute of Post Graduate Medical Education and Research, New Delhi, India

**Keywords:** atrial fibrillation, atrial myopathy, dapagliflozin, left atrial strain, rheumatic heart disease

Rheumatic heart disease (RHD) is a major cause of cardiovascular morbidity, with atrial fibrillation occurring in approximately 40% of patients.^1^^,^^2^ Chronic pressure or volume overload in RHD leads to left atrial (LA) stretching, inflammation, and extensive fibrosis, creating a substrate for atrial cardiomyopathy.^1^ Although standard RHD treatment focuses on antibiotic prophylaxis and valvular management, it does not address the underlying atrial remodeling. Dapagliflozin, a sodium-glucose cotransporter-2 (SGLT-2) inhibitor, has demonstrated anti-inflammatory and antifibrotic properties in heart failure and diabetes.^3^^,^^4^ This study aims to evaluate its impact on electrocardiographic (ECG) and echocardiographic markers of atrial myopathy in patients with rheumatic mitral valve disease (RMVD).

## Materials and Methods

This single-center, randomized, open-label study was conducted at the G.B. Pant Institute, New Delhi. The protocol was approved by the institutional ethics committee (F.1/IEC/MAMC/MD/MS(94/06/2022/No.460)) and was conducted in accordance with the Declaration of Helsinki and Good Clinical Practice guidelines.

### Participants

A total of 100 patients with moderate RMVD, stenosis, regurgitation, or both were enrolled. Exclusion criteria included pregnancy, hypertension, diabetes, chronic kidney disease, moderate to severe aortic disease, left ventricular systolic dysfunction (ejection fraction <50%), congenital heart disease, or coronary artery disease.

### Intervention

**Figure 1:**
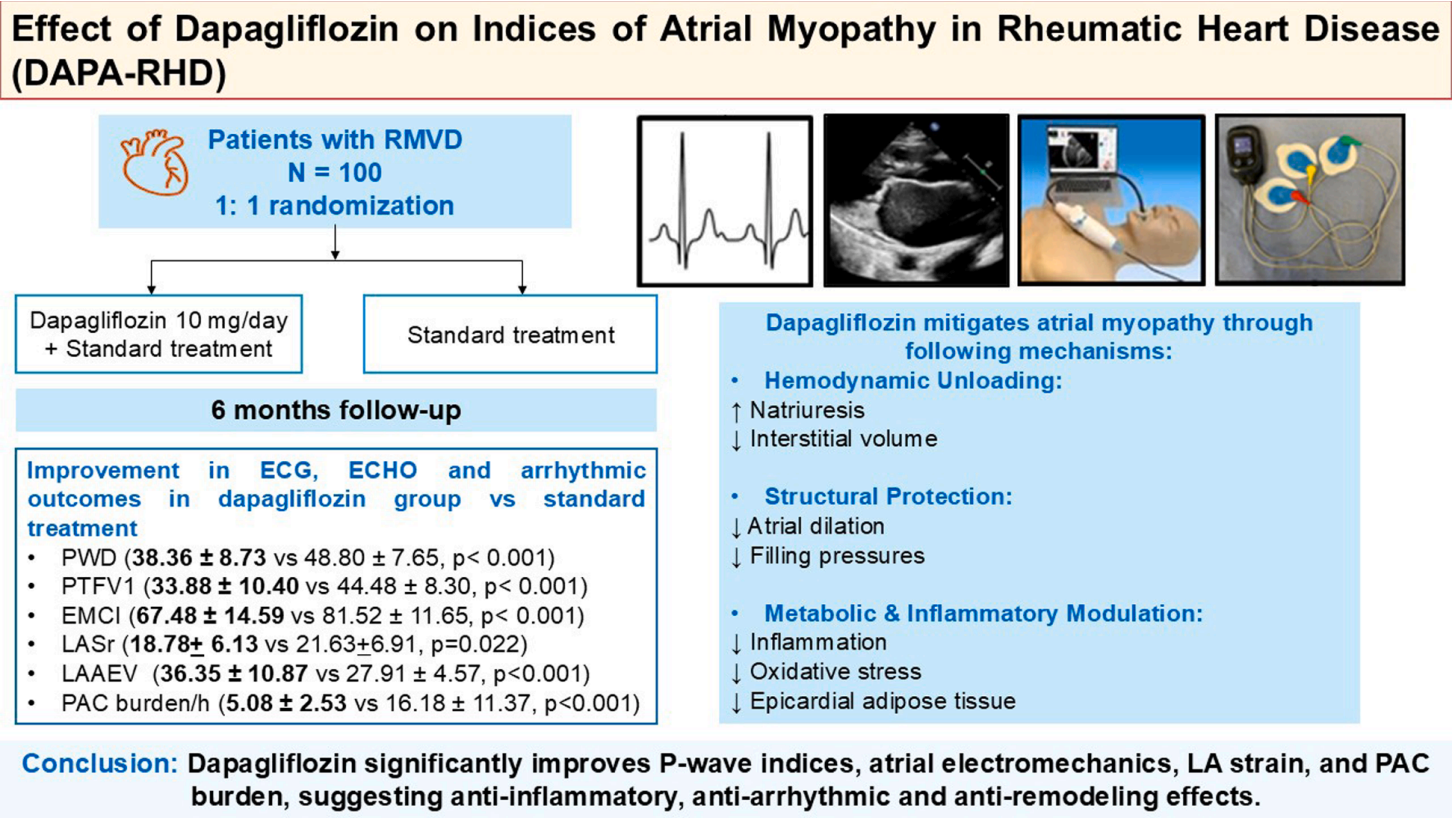
Effect of Dapagliflozin on Indices of Atrial Myopathy in Rheumatic Heart Disease Patients with rheumatic mitral valve disease (RMVD) were randomized 1:1 to receive either 10 mg/d of dapagliflozin plus standard medical treatment or standard treatment alone for 6 months. Indices of atrial myopathy, such as P-wave dispersion (PWD), P-wave terminal force in V_1_ (PTFV1), electromechanical coupling interval (EMCI), left atrial appendage emptying velocity (LAAEV), left atrial strain (LASr), and premature atrial complex burden, were measured at baseline and at 6 months. After 6 months, there was a significant improvement in ECG (PWD and PTFV1), ECHO (EMCI, LAAEV, and LASr), and arrhythmic (PAC burden) outcomes in the dapagliflozin group compared with standard treatment. The plausible mechanisms underlying the role of dapagliflozin in atrial myopathy include hemodynamic unloading, structural protection, and metabolic and inflammatory modulation. ECG = electrocardiogram; ECHO = echocardiographic; LA = left atrial; PAC = premature atrial complex.

Patients were randomized 1:1 to receive either 10 mg/d of dapagliflozin plus standard medical treatment or standard treatment alone for 6 months (Figure 1).

### Assessments

#### ECG markers

All ECGs were recorded at a paper speed of 50 mm/s and calibration of 1 mV/10 mm, and all measurements were performed by 2 experienced cardiologists blinded to the clinical data and study protocol. The P-wave duration was measured from a standard 12-lead digital ECG after amplification on a computer screen. The P-wave dispersion (PWD) in milliseconds was calculated by subtracting the minimum P-wave duration from the maximum P-wave duration in the 12-lead ECG.^5^ The PWD of >40 ms was considered significant.^4^ P-wave terminal force in V_1_ (PTFV1) was calculated by multiplying the duration (ms) and the depth (mm) of the downward deflection (terminal portion) of the P-wave in lead V_1_, and PTFV1 of >40 mm ms was considered abnormal.^5^ Twenty-four-hour ECG was recorded, and the premature atrial complex (PAC) burden per hour was calculated. We used the presence of ≥500 PACs/24 hours as a marker of potential atrial myopathy.^6^

#### Echocardiography

Atrial electromechanical coupling interval was measured via tissue Doppler imaging.^7^ LA strain (reservoir, conduit, and contractile) and left atrial appendage peak emptying velocity (LAAEV) were assessed using standard guidelines.^7^ Inactive LAA was defined as LAAEV <25 cm/s.

### Statistical Analysis

Statistical tests included the chi square test, the Student's *t*-test/Mann-Whitney test, and the Wilcoxon signed rank test. A 2-sided *P* value <0.05 was considered statistically significant.

## Results

Baseline characteristics were well-matched between groups; the mean age was 42.38 ± 8.61 years, and 77% (77 of 100) were women.

### ECG outcomes

After 6 months, the dapagliflozin group showed a significant reduction in PWD compared with the control group (38.36 ± 8.73 ms vs 48.80 ± 7.65 ms, *P* < 0.001) (Figure 1). Similarly, PTFV1 decreased significantly in the treatment group (33.88 ± 10.40 mm ms vs 44.48 ± 8.30 mm ms, *P* < 0.001). The number of patients with clinically relevant PWD (>40 ms) dropped from 78% (39 of 50) to 22% (11 of 50) after 6 months of dapagliflozin therapy.

### Echocardiographic and arrhythmic outcomes

#### Electromechanical coupling interval

There was a significant reduction in the dapagliflozin group (67.48 ± 14.59 ms vs 81.52 ± 11.65 ms, *P* < 0.001).

#### LA strain

Significant improvements were noted in the reservoir strain (21.63 ± 6.91 vs 18.78 ± 6.13, *P* = 0.022), conduit strain (−11.24 ± 4.66 vs −9.60 ± 1.79, *P* = 0.024), and contractile strain (−9.98 ± 3.78 vs −8.88 ± 1.83, *P* = 0.032) (Figure 1).

#### LAAEV

Peak emptying velocity increased significantly (36.35 ± 10.87 cm/s vs 27.91 ± 4.57 cm/s, *P* < 0.001) (Figure 1). At baseline, 40% (20 of 50) of patients had inactive LAA, which reduced to 10% (5 of 50) after 6 months of dapagliflozin therapy.

#### PAC burden

The treatment group saw a marked reduction in the number of patients having clinically relevant PAC burden (defined as ≥500 PACs/24 hours), which reduced from 38% (19 of 50) at baseline to 12% (6 of 50) at 6 months of dapagliflozin treatment (*P* = 0.001).

No significant adverse events or hospitalizations occurred during the study.

## Discussion

This study is the first to demonstrate that SGLT-2 inhibitors improve atrial myopathy indices in patients with RHD. Chronic inflammation and subsequent fibrosis in RHD lead to atrial structural and electromechanical remodeling and eventually atrial myopathy. In the context of atrial myopathy, P-wave indices suggest intra-atrial conduction abnormalities, LA strain indicates an anatomical substrate, and frequent PAC burden indicates an underlying pathology associated with enhanced automaticity. The risk of atrial fibrillation is greatest when all 3 domains are impacted. The addition of dapagliflozin to guideline-directed therapy significantly enhanced atrial electromechanics, shortened PWD, improved LA strain, increased LAAEV, and reduced PAC burden in moderate RMVD.

These results corroborate findings in diabetic cohorts, in which SGLT-2 inhibitors improved PWD, atrial electromechanical delay, and LA strain.^8^, ^9^, ^10^ Furthermore, the observed antiarrhythmic effects align with the DECLARE-TIMI 58 trial outcomes in type 2 diabetes mellitus.^4^

Dapagliflozin likely mitigates atrial myopathy through 3 primary mechanisms (Figure 1):

• Hemodynamic unloading: enhancing natriuresis and reducing interstitial volume.
• Structural protection: minimizing atrial dilation and filling pressures.
• Metabolic regulation: reducing inflammation, oxidative stress, and epicardial adipose tissue.

In conclusion, this trial provides foundational evidence that SGLT-2 inhibitors positively modulate the anatomical substrate, intra-atrial conduction, and automaticity characteristic of rheumatic atrial myopathy.

### Study Limitations

The study was a single-center pilot with a modest sample size and a brief (6-month) follow-up.

## Conclusions

In patients with moderate RMVD, dapagliflozin significantly improves atrial electromechanics, LA strain, and PAC burden. These findings suggest a potent anti-inflammatory, anti-remodeling, and antiarrhythmic role for SGLT-2 inhibitors in RHD, warranting larger trials to confirm their long-term clinical benefits.

## Funding Support and Author Disclosures

The authors have reported that they have no relationships relevant to the contents of this paper to disclose.
